# Wolbachia-Mediated Male Killing Is Associated with Defective Chromatin Remodeling

**DOI:** 10.1371/journal.pone.0030045

**Published:** 2012-01-23

**Authors:** Maria Giovanna Riparbelli, Rosanna Giordano, Morio Ueyama, Giuliano Callaini

**Affiliations:** 1 Department of Evolutionary Biology, University of Siena, Siena, Italy; 2 Illinois Natural History Survey, Institute of Natural Resource Sustainability, University of Illinois at Urbana-Champaign, Champaign, Illinois, United States of America; 3 Laboratory of Cell Biology, Department of Bioinformatics, Soka University, Hachioji, Tokyo, Japan; Louisiana State University and A & M College, United States of America

## Abstract

Male killing, induced by different bacterial taxa of maternally inherited microorganisms, resulting in highly distorted female-biased sex-ratios, is a common phenomenon among arthropods. Some strains of the endosymbiont bacteria *Wolbachia* have been shown to induce this phenotype in particular insect hosts. High altitude populations of *Drosophila bifasciata* infected with *Wolbachia* show selective male killing during embryonic development. However, since this was first reported, circa 60 years ago, the interaction between *Wolbachia* and its host has remained unclear. Herein we show that *D. bifasciata* male embryos display defective chromatin remodeling, improper chromatid segregation and chromosome bridging, as well as abnormal mitotic spindles and gradual loss of their centrosomes. These defects occur at different times in the early development of male embryos leading to death during early nuclear division cycles or large defective areas of the cellular blastoderm, culminating in abnormal embryos that die before eclosion. We propose that *Wolbachia* affects the development of male embryos by specifically targeting male chromatin remodeling and thus disturbing mitotic spindle assembly and chromosome behavior. These are the first observations that demonstrate fundamental aspects of the cytological mechanism of male killing and represent a solid base for further molecular studies of this phenomenon.

## Introduction

Several maternally inherited symbiotic bacteria are known to affect the reproductive biology of their host species by favouring female over male offspring. Mechanisms of sex-ratio distortion include the induction of parthenogenesis, feminization and male-killing of their arthropod host species [1]–[4]. The most dramatic form of sex-ratio distortion is male-killing in which bacteria pass from infected females to their progeny and selectively kill males they infect during embryogenesis, resulting in female-biased sex-ratios in their insect host.

Male killing bacteria belong to diverse taxa and are widespread among arthropods and common within insects [5]–[8]. in the genus *Drosophila*, male killing has been associated with the presence of maternally transmitted *Spiroplasma* and *Wolbachia* [9].

While the biology of male killing bacteria has been extensively studied at the ecological level, little is known about how bacteria and their hosts interact at the cellular and molecular level. Past works have examined male killing phenotypes induced by *Spiroplasma poulsonii* in *Drosophila willistoni* [10], *Drosophila nebulosa* [11], and *Drosophila melanogaster* [12], [13]. However, the underlying mechanisms that produce male-specific lethality remain unknown [4].

Cytological observations in *Drosophila willistoni* have suggested that male killing *Spiroplasma* interfere with the early development of embryos by affecting normal mitotic progression [10]. Whereas genetic evidence suggests that *Spiroplasma* can target some components of the male-specific sex-determination pathway [14]. Another male-killing organism, the γ-proteobacterium *Arsenophonus nasoniae* has been shown to induce male killing in the wasp *Nasonia vitripennis* by targeting maternally inherited centrosomes [15]. These findings suggest that male killing bacteria have evolved different modes of interaction with their insect hosts resulting in diverse pathways to embryo male death.

*Wolbachia* has been implicated in female biased sex-ratios in diverse arthropod host orders: from the arachnid Pseudoscorpiones [16], to the insect Coleoptera [17], Lepidoptera [18] and Diptera [19]. In *Drosophila bifasciata*, molecular systematic analysis and susceptibility to antibiotics have demonstrated that the male killing phenotype is not of viral origin [20], but is associated with a *Wolbachia* infection [21]. Moreover, it has also been shown that the male killing phenotype in *D. bifasciata* has high penetrance at low temperatures (18°C) and is reduced at high temperatures (26°C). This difference may be the result of reduced bacterial density at elevated temperatures [21].

This study aimed to examine the interaction between *Wolbachia* bacteria and *Drosophila bifasciata* by analysing early developmental stages of embryos obtained from crosses of infected females and uninfected males using fluorescent staining of both chromatin and microtubules.

Results from this study demonstrate that male embryos derived from *Wolbachia* infected *Drosophila bifasciata* females mated with uninfected males show severe defects of chromatin remodeling and spindle organization that result in abnormal mitoses and development failure. Our work leads us to conclude that this male-killing strain of *Wolbachia* plays a crucial role as a modulator of chromatin architecture and dynamics, pointing to the existence of a bacterial factor/s that regulate the chromatin remodeling of its eukaryotic host.

## Results

To characterize events associated with male embryo death in eggs of *Wolbachia*-infected *Drosophila bifasciata*, strain KOS10, we analyzed microtubule distribution and chromatin organization in embryos at various stages of development in order to score zygotic spindle formation, intravitelline and syncytial mitoses, cellular blastoderm formation, and later stages of embryogenesis.

To ascertain whether the primary block in development was due to sperm entry failure, we stained eggs collected from 30 minutes to 3 hours after egg deposition (AED) with an antibody directed against acetylated-α-tubulin, shown to recognize axonemal structures in cilia and flagella [22]. This antibody allowed the unambiguous tracing of the position of the sperm tail within the developing *Drosophila* embryo. Of the 273 embryos analyzed in this study, spanning from second meiosis to cellular blastoderm stage, 81% had a distinct sperm tail. This is a relatively low percentage in comparison to *Drosophila melanogaste* where a large majority of eggs deposited (>95%) have been shown to contain a detectable sperm tail that ends near the elongated nucleus in the anterior region of the egg [23]. To exclude the possible influence of *Wolbachia* bacteria on sperm entry into the egg, we also analyzed eggs obtained by the uninfected KOS1 strain. Of the 191 embryos examined, 83% contained a distinct sperm tail. The latter suggests that the reduced fertilization rate we observed was unrelated to the presence of *Wolbachia* bacteria but is a characteristic of this *Drosophila* population. Furthermore these observations indicate that failure of sperm entrance in the oocyte is not the primary cause of the early developmental block described in eggs obtained by KOS10 females.

### Female meiosis and gonomeric spindle formation in the *Wolbachia* background

**Figure 1:**
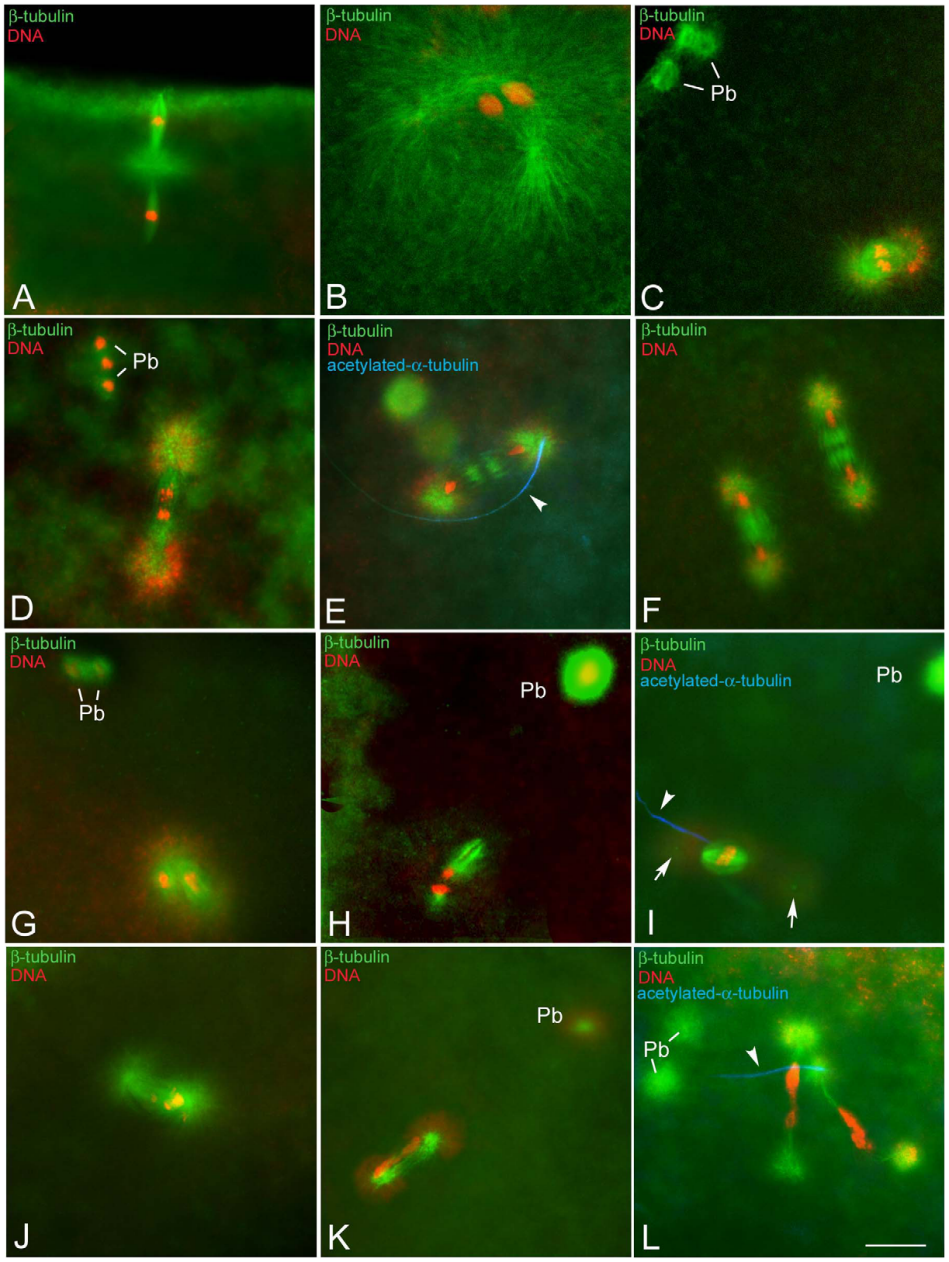
Formation of the gonomeric spindle in eggs obtained from KOS-10 *Wolbachia*-infected *Drosophila bifasciata* females. Eggs fixed 20 minutes AED were incubated with antibodies against β-tubulin (green), acetylated-α-tubulin (blue), and counterstained with Hoechst 33258 (red). (A) metaphase of the second meiosis: two anastral spindles, separated by a large microtubule aster, are aligned in tandem orthogonal to the egg surface. (B) Pronuclear apposition: male and female pronuclei come close within a large microtubule aster nucleated by two centrosomes derived by the male-basal body inherited centriole. (C) Metaphase of the first mitosis: the gonomeric spindle is formed by two closely apposed thin spindles sharing common poles; the parental complements move independently to the metaphase plate. (D) Anaphase of the first mitosis: parental complements start to migrate synchronously to the opposite poles. (E) Early telophase: midzone microtubules form the spindle midbody and daughter nuclei have reached the opposite poles. (F) Telophase of the second mitosis: two sister spindles hold four daughter nuclei. (G,H) Metaphase-like spindles: these gonomeric spindles appear disorganized and the two spindles that hold the parental complements are distant from each other. (I) Arrested anaphase spindles: chromosomes are arrested at the metaphase plate and centrosomes detached from the poles (arrows). (J) Abnormal anaphase: chromosomes move in a disorderly fashion to the opposite poles of the spindle. (K) Abnormal telophase: the spindle lacks a distinct midzone and chromatin forms large bridges. (L) Irregular second mitosis: spindles are unusually elongated and the chromosomes are stretched among the opposite poles. Arrowheads point to remnant of the sperm tail; Pb, polar bodies. Scale bar is 10 µm.

To determine whether the primary lesion leading to the formation of abnormal embryos was due to aberrant female meiosis, newly laid oocytes were stained for simultaneous visualization of microtubules and DNA. Oocytes scored 20 minutes AED (n = 53) had meiotic spindles of normal shape spanning from metaphase (Fig. 1A) to telophase of the second meiosis, where two tapered spindles aligned in tandem and oriented radially with respect to the oocyte surface. These spindles are typically anastral, but a monastral array of microtubules was found between them (Fig. 1A). The central aster contained a large accumulation of centrosomin (Cnn), confirming that the microtubules of the central asters were nucleated by bona fide centrosomal material (not shown). Female meiosis ends with the formation of four haploid chromosome complements that were aligned radially to the oocyte surface: the innermost haploid complement, the presumptive female pronucleus, moved toward the center of the egg where the male pronucleus was localized, whereas the other female complements moved to the egg surface to form polar bodies. These observations indicate that meiosis proceeded normally [24]–[26].

The assembly of the zygotic centrosome in wild-type eggs requires the recruitment of centrosomal components from the egg cytoplasm to a site adjacent to the sperm nucleus that corresponds to the basal body region [27]. To ascertain whether, in oocytes obtained by the KOS10 strain, the centrosomal components assembled around the sperm basal body to build a functional zygotic centrosome, we double stained newly fertilized eggs with anti-tubulin and Cnn. A distinct accumulation of Cnn was found associated with the sperm aster, suggesting that the recruitment of centrosomal components is normal in these eggs (not shown). Furthermore, double immunostaining with anti-tubulin antibodies and Hoechst dye for simultaneous visualization of microtubules and chromatin showed a prominent aster of microtubules in correspondence to the sperm nucleus (not shown). Therefore, defects in sperm centrosome assembly were not a primary cause of the early developmental block found in embryos of *Drosophila bifasciata*.

To determine whether abnormalities accounting for embryo death during early development may be due to defects occurring before or subsequent to the fusion of male and female pronuclei we analyzed the formation of the gonomeric spindle. Eggs collected 30–45 minutes AED were double stained for microtubules and DNA (n = 221; Table S1). In some eggs (n = 35; 15.8%) four haploid chromosome complements were found aligned radially to the egg surface, the innermost haploid complement being the presumptive female pronucleus together to a fifth anterior male nucleus. Other eggs (n = 25; 11.3%) displayed a large microtubule aster, the sperm aster, and two close nuclei, male and female (Fig. 1B). A gonomeric spindle composed of two closely apposed spindles holding separate paternal and maternal complements, organized during metaphase near the duplicated sperm centrosome (Fig. 1C). This spindle included the separate sets of parental chromosomes with a similar degree of condensation (Fig. 1C), as described during the fertilization of *Drosophila melanogaster* [28].

In 100 embryos (45.4%) the first mitosis progressed normally. At anaphase the parental complements segregated (Fig. 1D) and reached the opposite poles of the spindle during telophase (Fig. 1E). Acetylated-tubulin labeling revealed the persistence of the sperm tail at one pole of the zygotic spindle (Fig. 1E). When mitosis proceeded normally, two identical spindles were found within the inner cytoplasm (Fig. 1F). However, 10.8% (n = 24) of eggs scored during the first mitosis revealed abnormal figures spanning from disorganized gonomeric spindles, where the parental complements were held by widely separated spindles (Fig. 1G,H), to abnormal anaphase figures in which the chromosomes were arrested at the metaphase plate and centrosomes were detaching from the poles (Fig. 1I). We also found irregular anaphase/telophase configurations where chromosomes were scattered (Fig. 1J) or stretched (Fig. 1K) between the opposing poles. It was observed that 37 eggs (16.7%) scored after 30–45 minutes AED lacked a male pronucleus and a sperm tail, suggesting that fertilization failed in these eggs.

These observations led to the test of whether the improper positioning of the half parental complements in the gonomeric spindle could affect the normal progression of the second division. From 270 embryos collected 60–90 minutes AED (Table S1) 46 were during the second mitotic division: 39 of these embryos had chromosomes that were properly segregated (Fig. 1F), whereas in 7 embryos the chromatids had failed to segregate (Fig. 1L). The former embryos completed the second nuclear cycle, and formed two equally shaped mitotic spindles that synchronously supported chromosome congregation at the metaphase plate and sister chromatid movements to the opposite poles. The latter embryos had evident morphological abnormalities in both spindle organization and chromosome segregation and sister chromatids did not migrate properly in these spindles. The association of the sperm tail with the abnormal spindles during the first (Fig. 1I) and second (Fig. 1L) mitoses indicates that these figures were the result of problems that emerged after fertilization.

### Improper chromatin condensation during the syncytial mitoses is associated with defects of spindle organization

**Figure 2:**
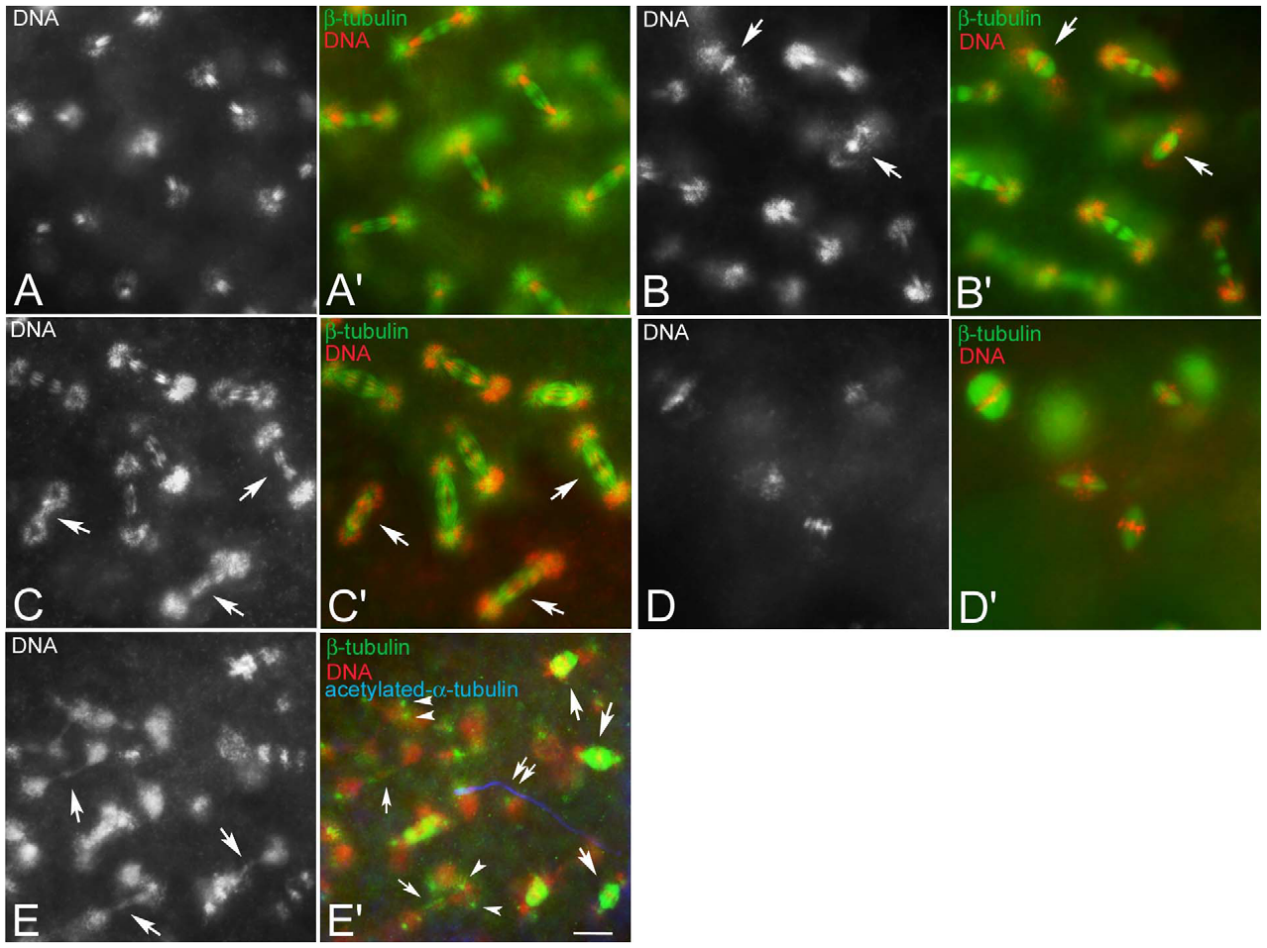
Improper chromosome segregation during the intravitelline mitoses. Black and white panels represent DNA staining alone; colour panels represent microtubules (green), DNA (red), and the sperm tail (blue). (A,A′) Detail of a normal embryo during early telophase of the seventh nuclear division cycle: sister chromatids have migrated at the opposite poles of the spindles to form daughter nuclei. Detail of embryos during early telophase (B,B′) or late anaphase (C,C′) of the seventh mitosis showing abnormal spindles: sister chromatids either do not separate and stay at the spindle equator (arrows in B) and the spindles are barrel-shaped (arrows in B′) or begin to separate, but do not migrate properly lagging in the spindle midzone or forming chromatin bridges (arrows in C), spindles do not show apparent defects at this stage (arrows in C′). (D,D′) Unfertilized eggs fixed 1 hour AED: note the anastral barrel spindles. (E,E′). Abnormal fertilized embryos fixed 1 hour AED: abnormal mitotic figures range from barrel-shaped biastral spindles (arrows in E′) to aberrant late telophase spindles in which the chromatin form dense bridges (larger arrows in E) and the centrosomes maintain their position closest to daughter nuclei (arrowheads in E′); late telophase spindles may be still recognized by the midbody remnants (small arrows in E′); double arrows in E′ point to the sperm tail. Bacteria associate with the poles of biastral spindles, but not with the poles of the anastral spindles. Scale bar is 4 µm.

The arrest phenotype in some embryos obtained from KOS10 females can be the result of defective steps in the nuclear division cycles. To test this possibility we examined embryos collected 60–90 minutes AED (Table S1). The main phenotypic class in this collection was represented by embryos found to be in the second mitosis (n = 46), as well as embryos in intravitelline mitoses (n = 149) (Fig. 2A,A′). However, further analysis revealed that some embryos that seemed to properly execute intravitelline mitoses had occasional defects consisting of spindles in which the progression through the nuclear division cycles was irregular. Defects such as barrel-shaped spindles with chromosomes in metaphase-like configuration (Fig. 2B,B′) and anaphase figures with lagging chromatids (Fig. 2C,C′) were encountered.

Of the eggs scored 60–90 minutes AED, 16.7% (n = 45) displayed dramatic abnormalities consisting of barrel shaped spindles that held chromosomes mostly oriented in a metaphase-like configuration (Fig. 2D,D′). Isolated centrosomes or free astral arrays of microtubules were never observed at this developmental stage. These figures, presumably, resulted from an early arrest due to the failure of sperm entry, as suggested by the absence of a sperm tail. The remaining embryos scored 60–90 minutes AED (n = 30; 11.1%) displayed biastral or monastral bipolar spindles as well as barrel shaped spindles that often fused together in larger microtubule aggregates in which the chromatin was irregularly packed (Fig. 2E,E′).

**Figure 3:**
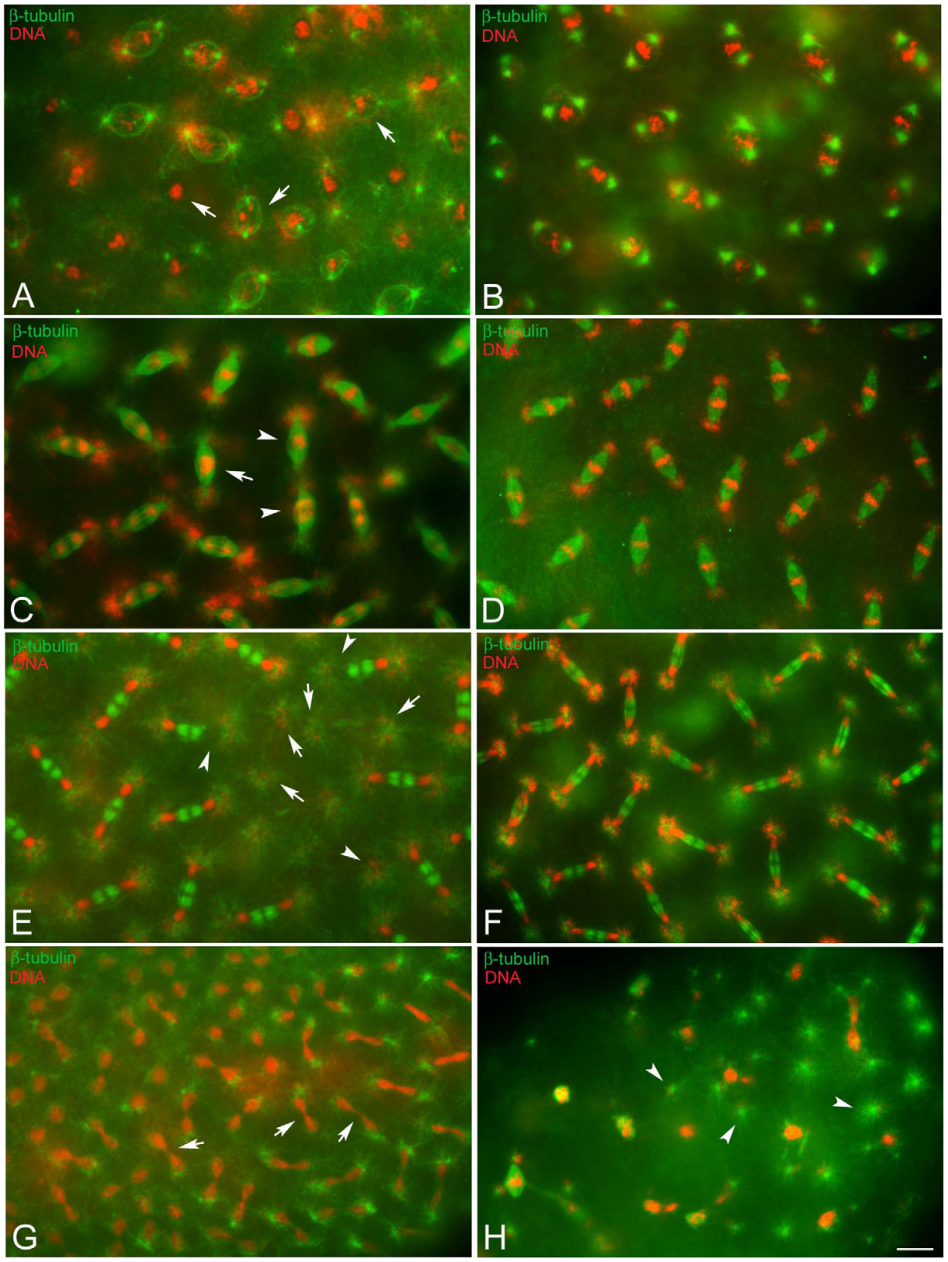
Defects of chromatin condensation and spindle organization during the syncytial blastoderm mitoses. Eggs fixed during the syncytial blastoderm stage and incubated with antibodies against β-tubulin (green), acetylated-α-tubulin (blue), and counterstained with Hoechst 33258 (red). (A,B) Prophase, (C,D) metaphase/anaphase, (E,F) telophase of the tenth nuclear division cycle: chromosomes have condensation defects (arrows in A, C) or do not migrate properly (arrowheads in C); telophase spindles without nuclei (arrows in E) or with only one daughter (arrowheads in E) are seen; although the poles of these spindles lack nuclei they have large asters to which bacteria are associated. (G) Detail of the anterior pole of an embryo during late telophase of the eleventh nuclear division cycle showing marked chromatin bridges (arrows). (H) Detail of an abnormal embryo showing irregular chromatin masses and free centrosomes (arrowheads). Scale bar is 4 µm.

Defects of pre-syncytial blastoderm embryos may be amplified subsequent to the migration of nuclei to the embryo surface and following syncytial blastoderm mitoses. To investigate this possibility the embryos were scored after nuclear migration during the tenth to thirteenth mitosis (Table S2). Of 336 embryos examined 3 hours AED 103 (30.6%) had some barrel shaped spindles or abnormal microtubule clusters and represent early developmental arrests or fertilization failures. Of the 233 embryos belonging to the syncytial blastoderm mitoses, 61% (n = 142) progressed normally through the nuclear division cycles, and 39% (n = 91) showed a wide range of mitotic defects. Defects in chromosome condensation were frequently observed. This is illustrated by the embryo in Fig. 3A where prophase spindles are organized around nuclei with differently condensed chromatin. This is in contrast to normal developing embryos where all prophase nuclei had similar entangled chromatin masses (Fig. 3B). Spindles that hold abnormally condensed chromatin also look abnormal. Some embryos had small surface areas with relevant asynchrony in the nuclear division cycles and metaphase and anaphase spindles observed near each other (Fig. 3C). During normal development, slight asynchrony in the nuclear division cycle and mitotic waves were observed. At the opposite poles of the embryo nuclear division cycles usually progressed gradually. However, central surface areas do not typically show asymmetric mitotic progressions (Fig. 3D). Some embryos obtained from KOS10 females also showed surface areas devoid of nuclei (Fig. 3E). In some cases telophase figures were observed in which the spindle held only one of the two expected daughters (Fig. 3E, arrows). This contrasts with the normal developing embryo where nuclei are evenly spaced (Fig. 3F). Another commonly observed defect was in chromosome segregation during anaphase and in the formation of chromatin bridges during telophase (Fig. 3G). Also observed were a lower number of embryos sharing abnormal DNA aggregates, irregular spindles and centrosomes that were found free in the cytoplasm or appeared detaching from the spindles (Fig. 3H).

### Chromatin condensation defects are amplified during cellularization and later stages of development

**Figure 4:**
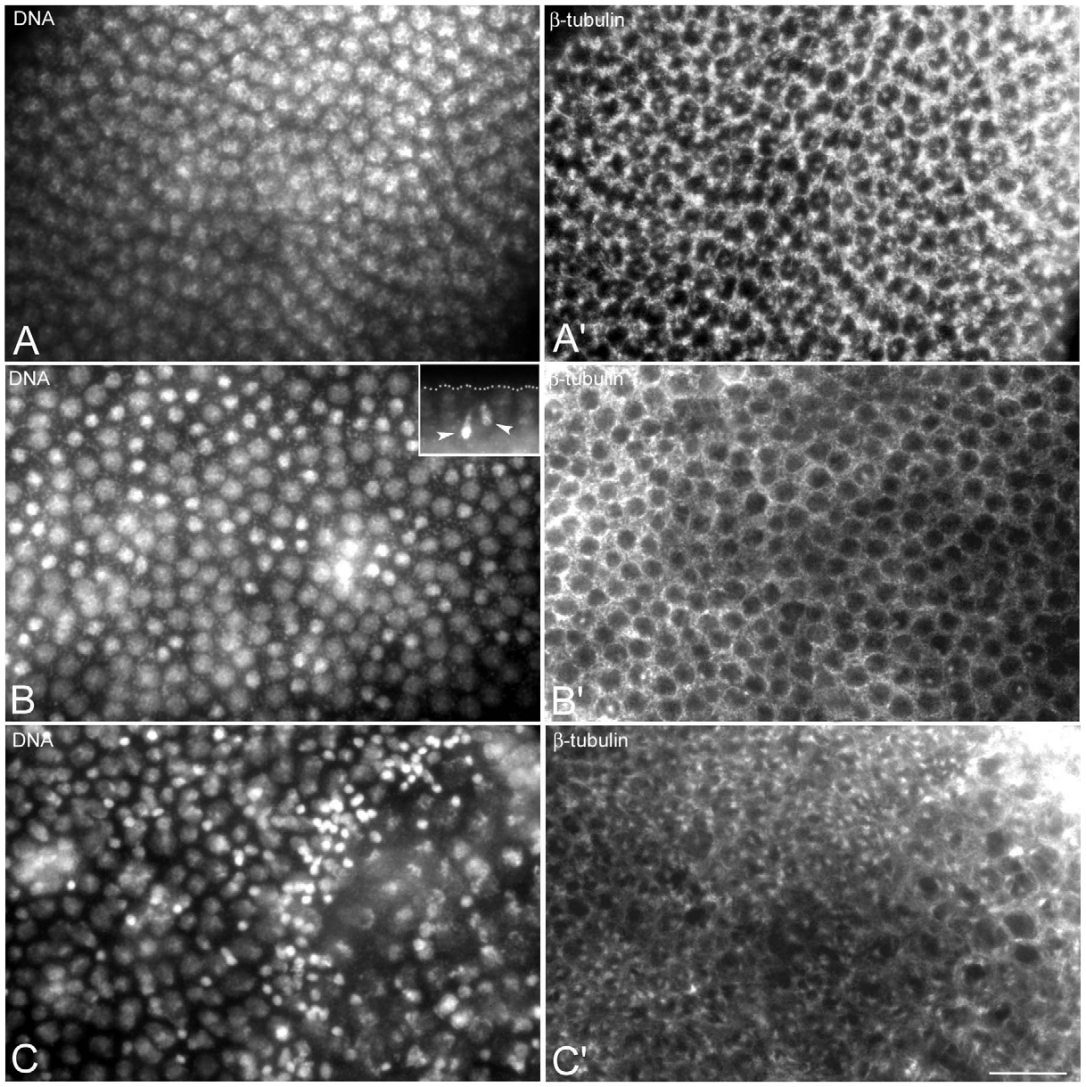
Nuclear condensation defects are amplified at cellularization. Embryos are stained for DNA (left panels) and microtubules (right panels). Cellular blastoderm is usually formed by evenly spaced nuclei at the same condensation stage and of the same size and dimensions (A) surrounded by honeycomb microtubular baskets (A′). Abnormal cellular blastoderms are characterized by small (arrow in B) or larger (arrows in C) clusters of picnotic nuclei scattered among normal looking blastoderm nuclei; the honeycomb microtubular baskets are often altered in these embryos (arrowheads in B′, C′). Scale bar is 15 µm.

To further characterize defects associated with the developmental arrest of embryos obtained by KOS10 females, embryos fixed 4–5 hours AED (Table S2), when the main phenotypic class was represented by cellular blastoderm, were also examined. Of the cellularizing embryos (n = 139), 61.1% (n = 85) displayed a normal blastoderm with interphase nuclei (Fig. 4A) surrounded by microtubular baskets (Fig. 4A′). The remnant cellularizing embryos (n = 54; 38.9%) displayed picnotic nuclei arranged in clusters or rows as well as dispersed within the interphase nuclei (Fig. 4B). Picnotic nuclei were associated with smaller microtubular baskets (Fig. 4B′) and were often seen to sink inward to the interior of the embryo (inset, Fig. 4B). Other embryos had large surface areas populated by irregular nuclei of various size and differeing chromatin condensation (Fig. 4C) that corresponded with abnormal arrangements of microtubular baskets (Fig. 4C′). Consequently, the distribution of surface nuclei was not uniform as seen in normal development.

**Figure 5:**
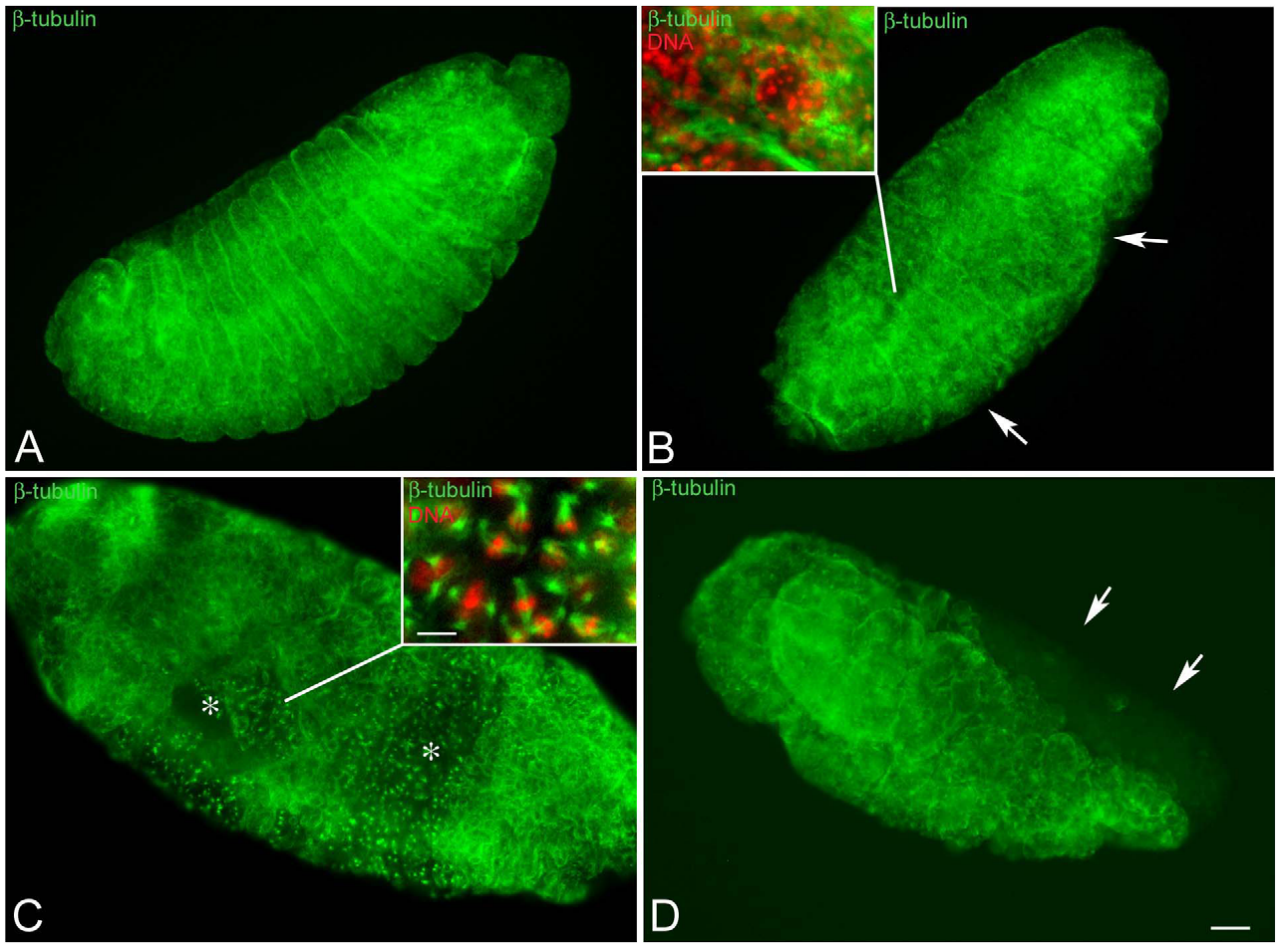
Embryos at later stages of development have highly defective areas. Embryos were fixed after 15–20 hours AED and stained for microtubules (green) and DNA (red). (A) Normal developing embryos in which the shortening of germ band is completed: segments are well evident. (B,C) Defective embryos after the retraction of the germ band: (B) the integrity of some segments is affected (arrows) and (C) large areas of the embryos are incompletely formed and still showed mitotic divisions (asterisks). (D) Detail of an embryo during germ band shortening: note the lack of differentiation of the anterior region of the body (arrows). Insets are details of the surface areas. Scale bar is 25 µm in the main panels and 7 µm in insets.

To ascertain whether the chromatin abnormalities observed in pre-blastoderm or cellularizing embryos may be inherited and/or amplified during further developmental stages, embryos fixed 10–20 hours AED (n = 292) were examined (Table S2). Of these: 9.5% (n = 28) were arrested very early during development, 18.5% (n = 54) were unfertilized, 8.9% (n = 26) were observed in various stages of development spanning from cellularization to later stages of gastrulation; 63.1% (184) displayed different stages of germ band extension-retraction. In this latter group, 61% (n = 112) formed what appeared to be normal segments (Fig. 5A), whereas the remnants 39% (n = 72) had significant abnormalities ranging from defects in segment formation (Fig. 5B), to failure to differentiate whole segments and the presence of unusual large areas of mitotic divisions (Fig. 5C), or incomplete body formation (Fig. 5D). These aberrations were linked to chromatin abnormalities that ranged from defects in morphology and condensation of sparse nuclei (inset, Fig. 5B) to nuclear and spindle abnormalities found in large areas of the embryo surface (inset, Fig. 5C).

### *Wolbachia* activity specifically targets chromatin of male embryos

**Figure 6:**
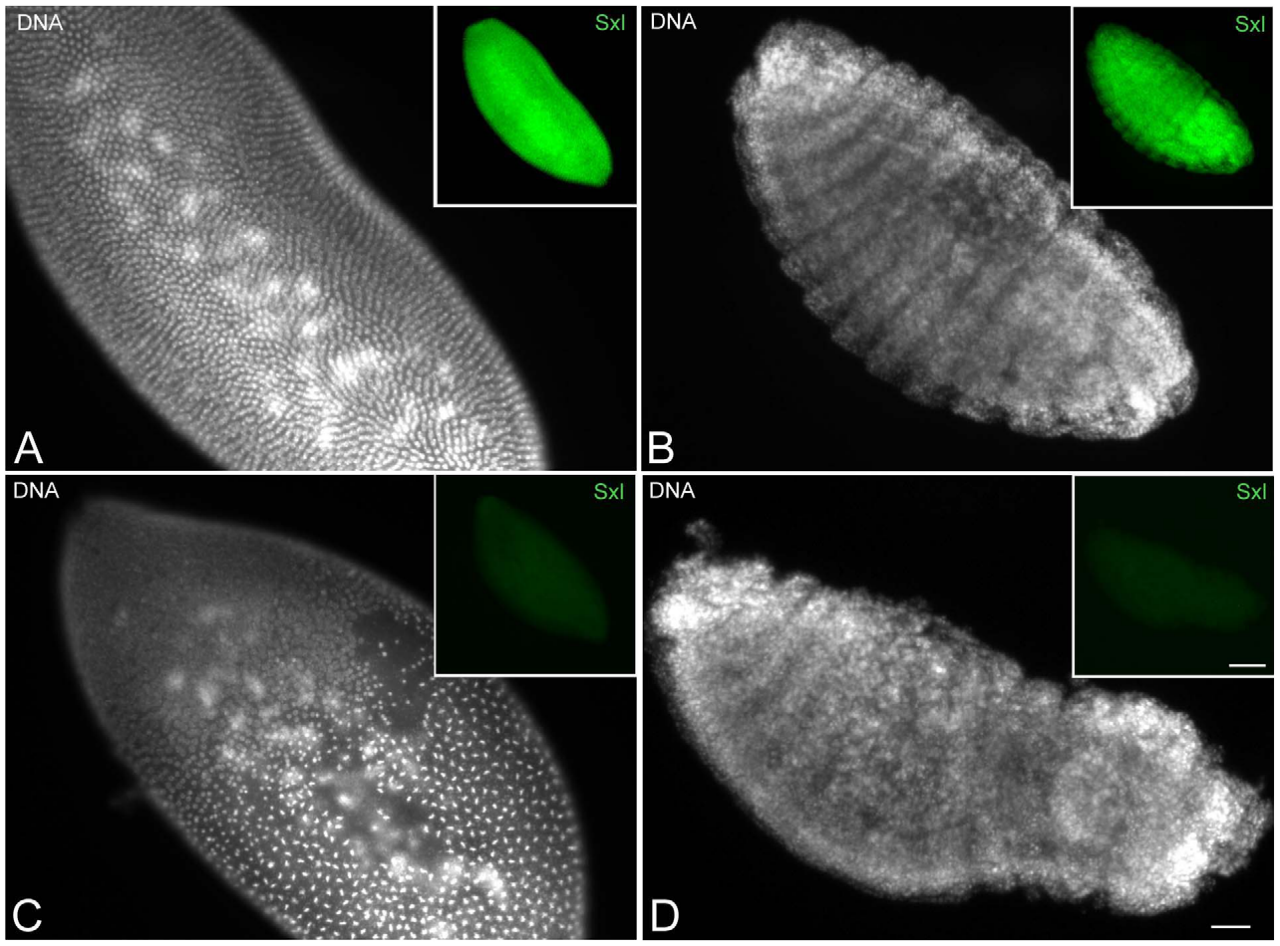
Sxl staining reveals that only male embryos have improper chromatin condensation. Embryos were stained for DNA (larger panels) and Sxl (insets, green). Sxl positive staining is associated with embryos showing normal blastoderm (A) and proper segmentation (B). Sxl antibody fails to stain embryos that have abnormal blastoderm (C) and improper segmentation (D). Scale bar is 25 µm in the main panels and 70 µm in insets.

Embryos with chromatin/spindle abnormalities, presumably leading to developmental arrest, represent about half of the embryos collected. KOS10 females lay eggs from which only viable females develop, therefore it is hypothesized that embryos with abnormalities in the nuclear progression cycles may represent males. To test this hypothesis we stained embryos fixed from 4 to 20 hours AED (Table S3) for DNA and the Sex-lethal protein (Sxl), a peptide required for sex determination in *Drosophila* and only expressed by the female line circa the beginning of nuclear cycle 12. Of the embryos collected (n = 197), 8.6% (n = 17) had an early arrested phenotype, 18.6 (n = 36) were unfertilized, and 73% (n = 144) were observed in various stages of development, spanning from last syncytial mitoses to germ-band shortening. Of the stained embryos, 81 embryos (41%) gave uniform staining with anti-Sxl antibody, whereas 63 (32%) lacked staining. If we assume that the early arrested embryos were male embryos, then the number of male is about 50% of the population of developing eggs. This observation is consistent with the expectation that *Sxl* is expressed only in females of *Drosophila bifasciata*. The Sxl gene is switched on in the syncytial blastoderm starting from the last nuclear division cycle, and embryos can be sexed unambiguously at beginning this time. Sxl expression was always found to be associated with the nuclei of embryos undergoing normal development (Fig. 6A,B), suggesting that females did not have developmental defects. By contrast, Sxl-negative embryos showed a variety of abnormalities ranging from defects in nuclear progression and chromatin condensation (Fig. 6C) to failure to form large embryonic surface areas during later developmental stages (Fig. 6D).

### Cytoplasmic asters are the landmark of early arrested embryos

**Figure 7:**
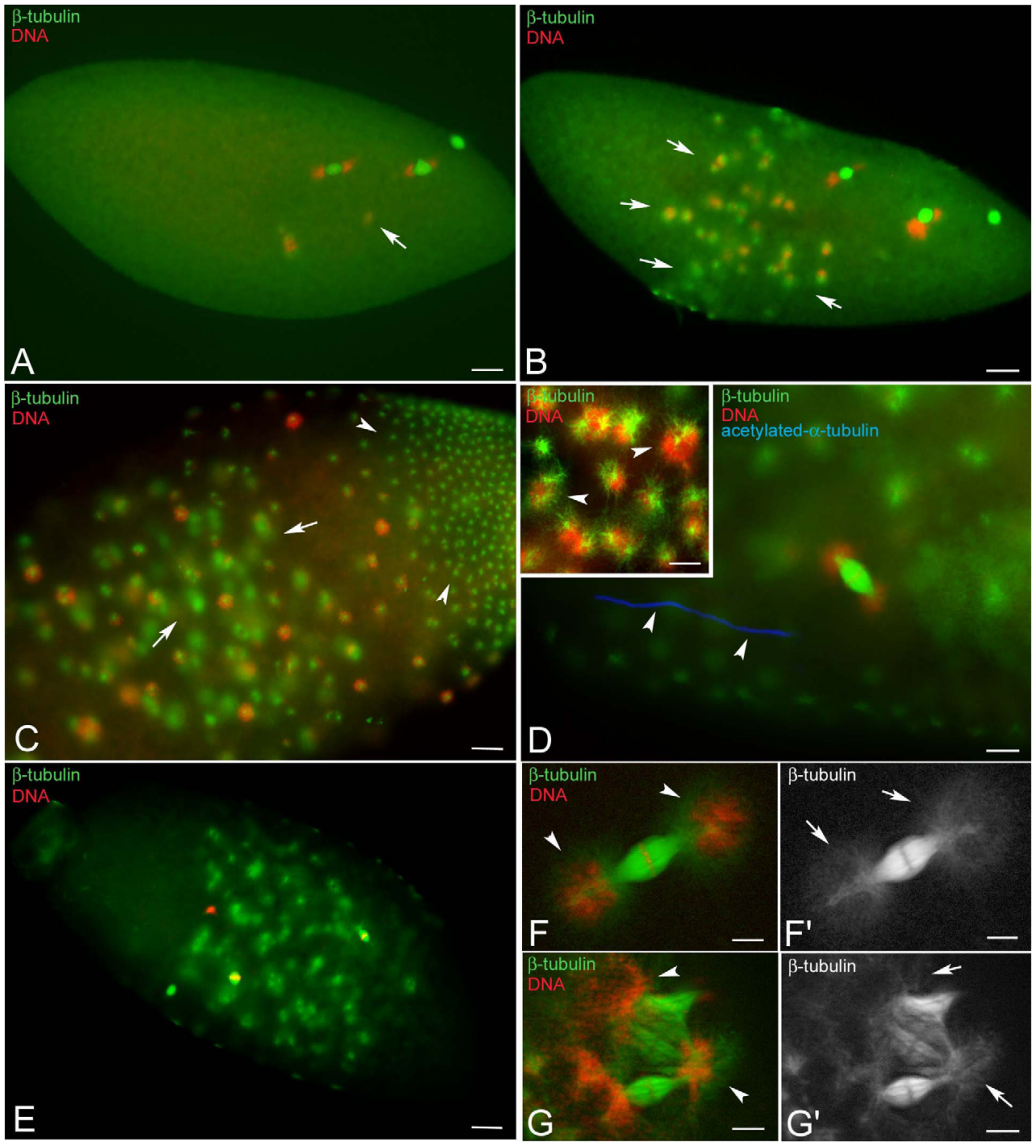
The early arrested phenotype is not due to *Wolbachia*. Eggs at different times AED were fixed and incubated with antibodies against β-tubulin (green), acetylated-α-tubulin (blue), and counterstained with Hoechst 33258 (red). Unfertilized eggs showed cytoplasmic asters that increase in number with age (A,B,C, arrows); old eggs also display smaller cortical asters (arrowheads). (E) Occasionally a sperm tail is found within the eggs that show cytoplasmic asters (arrowheads). Bacteria are associated with the cytoplasmic arrays of microtubules (arrowheads, inset D). (F) Unfertilized eggs from the uninfected KOS1 females also contain cytoplasmic asters. Bacteria are also observed at the poles of the meiotic-like spindles (arrowheads, F,G), where aster-like microtubule arrays are found (arrows, F′,G′). Scale bar is 25 µm in A,B,E; 15 µm in C; 4 µm in D; 5 µm in F,G.

Collection of embryos made at different times showed several non-developing eggs. A common characteristic was the presence of cytoplasmic asters that appeared early in development (Fig. 7A), increased in number with time (Fig. 7B), and reached high levels after 10 hours when large asters were present in the interior of the embryos and many small asters filled the cortical region (Fig. 7C). Presence of the sperm tail in rare eggs with cytoplasmic asters (Fig. 7D) suggested that the formation of these structures may be independent of sperm entrance. Unfertilized eggs obtained by KOS 1 females after 4–5 hours AED, also showed several free cytoplasmic asters in addition to metaphase-like barrel shaped spindles (Fig. 7E). This observation suggests that microtubule based asters also formed in the absence of bacteria and that they are a landmark of developmental failure.

Bacteria were found mainly in association with the astral microtubules (inset, Fig. 7D). However, some bacteria clusters were also found at the poles of the meiotic-like spindles (Fig. 7F,F′) and near the periphery of figures resulting from the fusion of two or three polar body spindles (Fig. 7G,G′). Many microtubules converged to the poles of these meiotic-like spindle structures (Fig. 7F′,G′), where bacteria clustered.

**Figure 8:**
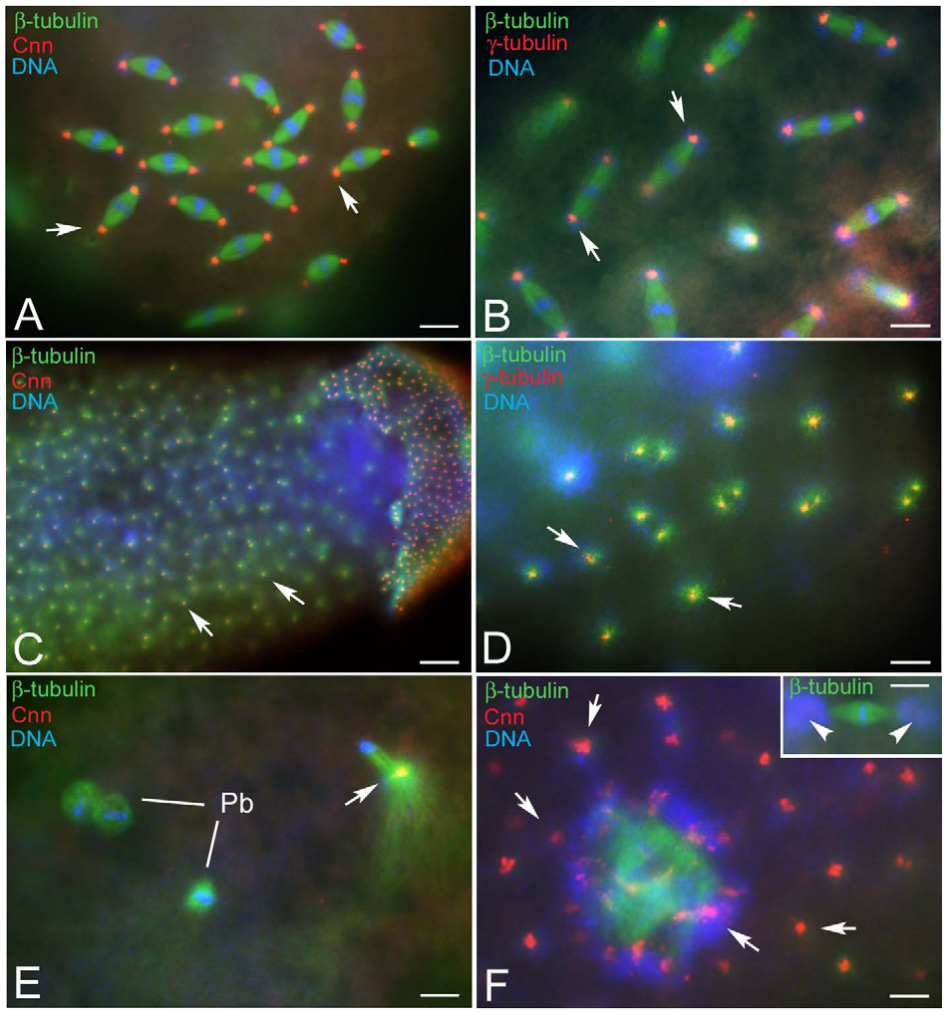
The focus of the cytoplasmic asters contains centrosomal components. Fertilized (A,B) and unfertilized (C–F) eggs obtained by KOS10 females are incubated with antibodies against β-tubulin (green); Cnn and γ-tubulin (red) and counterstained with Hoechst 33258 (blue). Cnn and γ-tubulin are evident at the spindle poles of normal developing embryos and at the focus of the cytoplasmic asters (arrows, A–D). (E) Detail of an unfertilized oocyte at the end of meiosis with a cluster of centrosomal material within the remnant of the central aster (arrow); Pb, polar bodies. (F) Cnn aggregates are found within the cytoplasm (arrows) and around the meiotic-like spindles (F, arrows), but not at their poles (inset F, arrowheads), despite the presence of microtubule asters and associated bacteria. Bar is 25 µm in C and 4 µm in all other panels.

To verify whether the astral arrays of microtubules were nucleated by true centrosomes, we looked at the localization of centrosomin (Cnn), a master protein required for centrosome organization and maturation [29] and γ-tubulin that dictates the nucleation of polarized microtubular arrays [30]. Antibodies against both of these centrosomal proteins recognized distinct spots at the poles of the mitotic spindles (Fig. 8 A,B). The foci of the cytoplasmic asters contained more or less large aggregates of Cnn (Fig. 8C) and γ-tubulin (Fig. 8D) suggesting that these microtubule arrays were nucleated by discrete centrosomal material. At the end of meiosis, unfertilized oocytes can retain the large central aster that characterizes the female meiotic spindle. A Cnn aggregate was found in the remnant of the central aster (Fig. 8E). Surprisingly, thin clusters of Cnn material surrounded the periphery of the multi-spindle structures (Fig. 8F), where bacteria accumulated. By contrast, no evidence of Cnn material was associated with the poles of isolated meiotic-like spindles (inset, Fig. 8F), despite the observation that some microtubule bundles were found in aster-like disposition. These findings suggest that these microtubules are not nucleated at the spindle poles and may have an acentrosomal origin.

## Discussion

It has been shown that female insects infected by some species of endosymbiotic bacteria, including the genera *Wolbachia* and *Spiroplasma* produce very strong female-biased progeny when mated with uninfected males [7]. However, despite the key role of these microorganisms, very little is known about the cellular mechanisms involved in this process. This study is a cytological analysis of the early development of eggs obtained by crosses of *Wolbachi*a infected *Drosophila bifasciata* females with uninfected males.

Previous studies [6], [20], [31], [32] showed that the sex ratio bias distortion observed in *Drosophila bifasciata* crosses is a result of a reduced male hatching rate compared to control uninfected females. However, there is little precise knowledge regarding the timing of male death. Earlier observations in *Drosophila willistoni* and *Drosophila melanogaster* infected by *Spiroplasma* suggest that the triggering of embryo defects occur at any point of development, from intravitelline mitoses to germ band shortening, with death occurring even in later stages [10], [12]. Findings using *Drosophila nebulosa* infected with *Spiroplasma* have shown that male embryos arrest development between stages 11 and 13 [11]. The cause for the discrepancy between these observations remains unclear, but may be a host dependent phenotype. Using transfection experiments, it has been demonstrated, that the expression intensity of the *Spiroplasma*-induced male killing varies depending on the host background within a single species [33].

Analysis of our data revealed that there is a gradual increase of embryonic death during early development. Given that the percentage of unfertilized eggs and embryos arrested during the early intravitelline mitoses was almost constant through the developmental stages, it is possible that some abnormal embryos escaped detection continue to develop and were counted at each stage. It is possible that male-killer bacteria act a specific time, but the effect of the action might be prolonged over the developmental time period. Alternatively, a threshold effect, requiring a critical concentration of bacterial product/s within the embryo, could be implicated to explain the phenotype we observed in *Drosophila bifasciata* females infected with *Wolbachia* where the arrest of male embryos was observed at any stage of development. This is supported by the finding that mitotic abnormalities occurring during blastoderm formation and gastrulation affect separate clusters of nuclei, indicating that mitotic problems occurred independently in different regions of the embryo- The effect is first seen in isolated nuclei that in turn transmit their abnormalities to progeny nuclei where the problems are amplified, forming clusters of irregularly condensed nuclei. Nuclei in these areas tend to be picnotic and sink inward suggesting the existence of a checkpoint monitoring incorrect chromatin organization.

It has been demonstrated that reducing bacterial density within eggs following exposure to elevated temperature leads to the decrease of male killing effects [6]. Consistently, mothers with a higher density of bacteria have more female-biased offspring sex-ratio [34]. Moreover, a *Spiroplasma* strain that normally causes early male killing also induces late male mortality in larvae and pupae in the offspring of young females [35], suggesting a correlation with maternal host age. The stage 13 male embryos we observed with no apparent defects or only small abnormalities could account for a class of late male-killing phenotypes, likely obtained by young females. Consistent with this hypothesis these embryos were found in batches in which there were large numbers of unfertilized eggs produced more frequently by young females. Intracellular density of the endosymbionts also represents a limiting factor for the intensity of cytoplasmic incompatibility and the strength of the phenotype induced [36]–[41].

Although male killing almost invariably results in death of the male embryos, past studies have suggested that the mechanisms potentially involved in this process do not require the Y chromosome as a specific target, since XXY females survive, and transmit the infection to their progeny [42]. Rather, it appears that flies with a single X chromosome are killed regardless of their phenotypic sex [43]. This indicates that elements of the dosage compensation system may represent the target of male-killing bacteria. This system compensates the unbalance between the unique X chromosome of males and the two chromosomes of females by augmenting X-chromosome linked transcription of the X-linked genes in males. Consistently, mutants of the *tra* gene that bear two X chromosomes are not killed by *Spiroplasma* and develop as somatic males [42]. Genetic analysis showed, that *Spiroplasma* failed to kill males of *Drosophila melanogaster* lacking any of the components of the dosage compensation complex [14], pointing to the requirement of a functional dosage compensation complex for the expression of bacteria mediated male-killing. Consistently, the expression of a doublesex homologue is altered in sexual mosaics of *Ostrinia scapulalis* moths infected with *Wolbachia* [44]. However, this does not appear to be the sole mechanism of male-killing activity. Male-killing *Wolbachia* have been described in both male and female heterogametic species suggesting that the dosage compensation pathway may not be the single focus of male-killing activity [5]. Moreover, the observation that the action of *Wolbachia* in the lepidopteran *Hypolimnas* can also contribute to male death in larval stages indicates that male-killing is not solely dependent on a process confined to embryos but may encompass the sex determination system that occurs early in embryogenesis [45]. Our finding of an earlier embryonic lethal phase, at the time of parental chromosome segregation during the first and second mitoses not related to the downstream male-specific developmental pathways that are activated at later developmental stages [46], support the possibility of multiple targets for male-killing bacteria in addition to or as alternatives to the elements of the sex determination machinery. The observation that the bacterium *Arsenophonus nasoniae* inhibits the formation of maternal centrosomes thus leading to mitotic defects associated with embryo death in *Nasonia*, indicates that bacteria have evolved different mechanisms for inducing male-killing in arthropods [15].

Although the activity of *Wolbachia* on chromatin remodeling during male-killing in *Drosophila bifasciata* appears unquestionably based on the chromosome segregation/condensation defects we observed, it remains unclear whether the spectrum of defects we observed affect all the chromosomes within the same spindle, why only the chromatin of male embryos is affected, and which specific mechanisms are involved.

Chromosome condensation and segregation during cell division is mainly dependent on the regulation of chromatin organization [47], [48]. Defective chromatin remodeling of the X chromosome might lead to other global effects such as improper replication and tangling of daughter DNA strands, ultimately causing failure of chromatid separation and bridging at anaphase/telophase resulting in abnormalities of the whole spindle. Accordingly, widespread mitotic defects leading to death of hybrid females produced from *Drosophila simulans* mothers and *Drosophila melanogaster* fathers are induced by separation failure of the paternally inherited X chromatids that are improperly packed as heterochromatin in the maternal background [49]. The improper condensation of the paternal chromosomes contributes to the chromatin bridging responsible for embryo death during the cytoplasmic incompatibility crosses [3], [50]–[53]. Therefore, the findings of a close relationship between the male-killing phenotype and chromatin remodeling may point to a similar mechanism for male-killing and cytoplasmic incompatibility, suggesting that cytoplasmic incompatibility and male-killing may involve similar targets or may follow a similar pathway as in the *Drosophila* system. This is supported by the finding that the transfer of some *Wolbachia* strains between *Drosophila* species results in a transition from cytoplasmic incompatibility to male-killing, indicating that these *Wolbachia* induced phenotypes share common molecular mechanisms [54].

Although our results on *Drosophila bifasciata* suggest a strong correlation between the male killer *Wolbachia* and chromatin remodeling in male embryos, a critical issue is how *Wolbachia* might affect chromatin remodeling. Nucleosomes are pivotal in establishing the architecture of chromosomes. Interactions between nucleosomes result in the formation of fibers that permit further packaging and a more compact chromosomal structure [55], [56]. This process is mainly dependent on linker histones that stabilize the interaction between nucleosomes and chromatin fibers [57]. Histone-modifying enzymes or chromatin-remodeling complexes can be targeted to specific promoters by gene-specific or general transcription factors, *Wolbachia* may interfere with any of the transcription pathways that regulate some of these processes, thus locally altering the structure or positioning of nucleosomes, histone-modifying enzymes or chromatin-remodeling enzymes. Accordingly, it has been demonstrated that *Wolbachia*-mediated cytoplasmic incompatibility is associated with impaired histone deposition in the male pronucleus [58].

The phenotype we observed in older unfertilized eggs is reminiscent of some *Drosophila* mutants that display high numbers of large cytoplasmic asters at the embryo surface [59]–[63]. The formation of these structures is hypothesized to be a secondary effect of centrosome detachment from the spindle poles. Some *Drosophila bifasciata* eggs we examined did not contain a sperm tail, suggesting that they were unfertilized and, therefore, lacking paternal centriole contribution needed to assemble the first zygotic centrosome. Since the focus of the asters contains centriolar and centrosomal markers, the question remains as to how centrioles/centrosomes can form *de novo* in the *Drosophila bifasciata* unfertilized egg. The trivial answer may be that in these eggs, as in parthenogenetic eggs, there is a mechanism leading to the *de novo* formation of centrosomes. This could be an intrinsic property of the *Drosophila* egg, since it has been shown that the cortical microtubule cytoskeleton of mature wild-type eggs was reorganized upon activation and expressed a transient assembly of cortical asters [64], like those observed in *Drosophila bifasciata*.

In developing embryos, *Wolbachia* is known to concentrate around the astral microtubules nucleated by distinct centrosomes [65], [66]. Interestingly, the symbiotic bacteria in *Drosophila bifasciata* were seen to associate with the free cytoplasmic asters nucleated by the maternal centrosomes, but also accumulated at the poles of the acentrosomal spindles derived by the acentriolar meiotic complements. The reason for this unexpected association is unknown. These spindles are, however, unusual in their architecture, since they present aster-like structures at the poles in the absence of recognizable centrosomes.

Our results on the effects of *Wolbachia-*induced male-killing in *Drosophila bifasciata* suggest a strong correlation between the male-killer *Wolbachia* and chromatin remodeling in male embryos. A critical issue remaining is whether the abnormalities in nuclear divisions we observed reflect a direct action of a *Wolbachia* product in some aspect of chromosome structure and function or whether they arise by an indirect mechanism that is triggered by *Wolbachia*.

## Materials and Methods

### *Drosophila* stocks

The lines of *Drosophila bifasciata* KOS-1 and −10 used in this work were established from iso-females collected in May, 2004 at Matsuhime pass (N 35°43′38.5″E 138°56′59.4″, altitude 1250 m). The *Wolbachia* infection was confirmed by PCR amplification of 16SrRNA, *ftsZ*, and *wsp* genes. The KOS-1 and −10 are *Wolbachia* uninfected and infected line, respectively. KOS-10 line has been kept by crossing males of the KOS-1 line after these lines were established and reared over circa 50 generations, thus the genetic background of these lines is nearly equal. The flies were reared on standard *Drosophila* medium at 17°C. No specific permissions were required for research on *Drosophila bifasciata* eggs/embryos as is not a protected species.

### Antibodies

We used the following antibodies: mouse anti-β-tubulin (Boehringer, Mannheim UK; 1∶200); rat YL1/2 directed against tyrosinated-α-tubulin (Harlan Sera-Lab, 1∶20); mouse anti-γ-tubulin (Sigma- Aldrich, 1∶100); mouse anti-acetylated-α-tubulin (Sigma-Aldrich; 1∶100); rabbit anti-centrosomin (Cnn, 1∶400; [67]); mouse anti-Sex lethal protein (Sxl) M18 and M114 clones (Developmental Studies Hybridoma Bank, Iowa University). The secondary antibodies Alexa Fluor 488 and 555 (InVitrogen) anti-mouse, anti-rabbit and anti-rat were used at 1∶800 dilutions.

### Egg collection

Embryos were collected from 4- to 5-day-old flies placed on small agar-yeast plates. Eggs were fixed at 20 minutes to obtain meiotic stages or held for 30–45 minutes before fixation to examine gonomeric spindle formation and early mitotic divisions. Eggs that developed one to three hours after collection yielded embryos that were sufficiently young to contain intravitelline mitoses and syncytial stages mainly at nuclear cycles 8–11, and some older embryos. Embryos at cellularization and early gastrulation stages were obtained by waiting 4–5 hours, whereas later developmental stages were obtained by eggs allowed to develop 10 to 20 hours.

### Fixation and staining

Eggs were dechorionated in a 50% bleach solution for 2–3 minutes and rinsed in distilled water. Eggs were transferred to a heptane/methanol solution (1∶1) and the vitelline membrane was removed by vigorous agitation for 3 min. Eggs were then fixed 10 min in cold methanol, washed in PBS and incubated for 1 h in PBS containing 0.1% bovine serum albumin (BSA).

For simultaneous localization of microtubules and γ-tubulin the eggs were incubated overnight at 4°C with the anti-γ-tubulin antibody, then the YL1/2 antibody was added and the incubation was allowed to proceed for 2 hours at room temperature. For simultaneous localization of the sperm tail and α-tubulin the eggs were incubated 3 hours at room temperature in both anti-acetylated-α-tubulin and YL1/2 antibodies. For double staining of microtubules and Sxl or Cnn the eggs were incubated overnight at 4°C with the specific antisera and then with anti-α or anti-β tubulin antibodies for 3 hours at room temperature.After washing in PBS–BSA, the eggs were incubated for 1 h with the appropriate secondary antibodies. Controls of the secondary antibodies alone were done for all staining. After washing in PBS–BSA, in all cases DNA was visualized by 3–4 minute incubation in Hoechst 33258. Samples were mounted in small drops of 90% glycerol in PBS.

### Image acquisition and presentation

The fluorescent images were taken using an Axio Imager Z1 (Carl Zeiss) microscope equipped with an HBO 50-W mercury lamp for epifluorescence and with an AxioCam HR cooled charge-coupled camera (Carl Zeiss). Image projections of embryos were done with Z-stack and Extended Focus modules (Carl Zeiss). Gray-scale digital images were collected separately and then pseudocolored and merged using Adobe Photoshop 5.5 software (Adobe Systems).

## Supporting Information

Table S1 Developmental defects observed in early embryos obtained by *Drosophila bifasciata* KOS10 females.
(DOC)

Table S2 Developmental defects observed in late embryos obtained by *Drosophila bifasciata* KOS10 females.
(DOC)

Table S3 Anti-Sxl staining of embryos collected 4–20 hours AED by *Drosophila bifasciata* KOS10 females.
(DOC)

## References

[1] Stouthamer R, Breeuwer JAJ, Hurst GDD (1999). *Wolbachia pipientis*: microbial manipulator of arthropod reproduction.. Annu Rev Microbiol.

[2] Stevens L, Giordano R, Fialho RF (2001). Evolution, systematics and ecology of *Wolbachia* infections in arthropods.. Annu Rev Ecol Syst.

[3] Serbus LR, Casper-Lindley C, Landmann F, Sullivan W (2008). The genetics and cell biology of *Wolbachia*-host interactions.. Annu Rev Genet.

[4] Werren JH, Baldo L, Clark ME (2008). *Wolbachia*: master manipulators of invertebrate biology.. Nat Rev Microbiol.

[5] Hurst GDD, Jiggins FM (2000). Male-killing bacteria in insects: mechanisms, incidence, and implications.. Emer Inf Dis.

[6] Hurst GDD, Hurst LD, Majerus MEN, O'Neill SL, Hoffmann AA, Werren JH (1997). Cytoplasmic sex-ratio distorters.. Influential Passengers.

[7] O'Neill SL, Hoffmann AA, Werren JH (1997). Influential passengers: inherited microorganisms and arthropod reproduction..

[8] Bourtzis K, Miller TA (2003). Insect symbiosis..

[9] Montenegro H, Solferini VN, Klaczko LB, Hurst GGD (2005). Male-killing *Spiroplasma* naturally infecting *Drosophila melanogaster.*. Insect Mol Biol.

[10] Counce SJ, Poulson DF (1962). Developmental effects of the sex-ratio agent in embryos in *Drosophila willistoni*.. J Exp Zool.

[11] Bentley JK, Veneti Z, Heraty J, Hurst GDD (2007). The pathology of embryo death caused by the male-killing *Spiroplasma* bacterium in *Drosophila nebulosa.*. BMC Biology.

[12] Tsuyichiya-Omura S, Sakaguchi B, Koga K, Poulson DF (1985). Morphological features of embryogenesis in *Drosophila melanogaster* infected with male-killing spiroplasma.. Zool Sci.

[13] Kuroda K, Shimada Y, Sakaguchi B, Oishi K (1992). Effects of Sex ratio (SR) *Spiroplasma* infection of *Drosophila* primary embryonic cultured cells and on embryogenesis.. Zool Sci.

[14] Veneti Z, Bentley JK, Koana T, Braig HR, Hurst GDD (2005). A functional dosage compensation complex required for male killing in *Drosophila*.. Science.

[15] Ferree PM, Avery A, Azpurua J, Wilkes T, Werren JH (2008). A bacterium targets maternally inherited centrosomes to kill males in *Nasonia*.. Curr Biol.

[16] Zeh DW, Zeh JA, Bonilla MM (2005). *Wolbachia*, sex ratio bias and apparent male killing in the harlequin beetle riding pseudoscorpion.. Heredity.

[17] Fialho RF, Stevens L (2000). Male-killing *Wolbachia* in a flour beetle.. Proc R Soc Lond B.

[18] Jiggins FM, Hurst GD, Schulenburg JH, Majerus ME (2001). Two male-killing *Wolbachia* strains coexist within a population of the butterfly *Acraea encedon*.. Heredity.

[19] Dyer KA, Jaenike J (2004). Evolutionarily stable infection by a male-killing endosymbiont in *Drosophila innubila*: molecular evidence from the host and parasite genomes.. Genetics.

[20] Magni GE (1953). ‘Sex-ratio’: a non-Mendelian character in *Drosophila bifasciata.*. Nature.

[21] Hurst GDD, Johnson AP, Hinrich JG, Schulenburg VD, Fuyama Y (2000). Male-killing *Wolbachia* in *Drosophila*: A temperature-sensitive trait with a threshold bacterial density.. Genetics.

[22] Piperno G, Fuller MT (1985). Monoclonal antibodies specific for an acetylated form of alpha-tubulin recognize the antigen in cilia and flagella from a variety of organisms.. J Cell Biol.

[23] Riparbelli MG, Callaini G (2010). Detachment of the basal body from the sperm tail is not required to organize functional centrosomes during *Drosophila* embryogenesis.. Cytoskeleton.

[24] Riparbelli MG, Callaini G (2005). The meiotic spindle of the *Drosophila* oocyte: the role of centrosomin and the central aster.. J Cell Sci.

[25] Riparbelli MG, Callaini G (1996). Meiotic spindle organization in fertilized *Drosophila* oocyte: presence of centrosomal components in the meiotic apparatus.. J Cell Sci.

[26] Endow SA, Komma DJ (1998). Assembly and dynamics of an anastral:astral spindle: the meiosis II spindle of *Drosophila* oocytes.. J Cell Sci.

[27] Riparbelli MG, Whitfield WG, Dallai R, Callaini G (1997). Assembly of the zygotic centrosome in the fertilized *Drosophila* egg.. Mech Dev.

[28] Callaini G, Riparbelli MG (1996). Fertilization in *Drosophila melanogaster*: centrosome inheritance and organization of the first mitotic spindle.. Dev Biol.

[29] Zhang J, Megraw TL (2007). Proper recruitment of gamma-tubulin and D-TACC/Msps to embryonic *Drosophila* centrosomes requires Centrosomin Motif 1.. Mol Biol Cell.

[30] Oakley BR (2000). Gamma-Tubulin.. Curr Top Dev Biol.

[31] Ikeda H (1970). The cytoplasmically-inherited ‘sex-ratio’ condition in natural and experimental populations of *Drosophila bifasciata*.. Genetics.

[32] Veneti Z, Toda MJ, Hurst GGD (2004). Host resistance does not explain variation in incidence of male-killing bacteria in *Drosophila bifasciata*.. BMC Evol Biol.

[33] Kageyama D, Anbutsu H, Shimada M, Fukatsu T (2009). Effects of host genotype against the expression of spiroplasma-induced male killing in *Drosophila melanogaste.*. Heredity.

[34] Dyer KA, Minhas MS, Jaenike J (2005). Expression and modulation of embryonic male-killing in *Drosophila innubila*: opportunities for multilevel selection.. Evolution.

[35] Kageyama D, Anbutsu H, Shimada M, Fukatsu T (2007). *Spiroplasma* infection causes either early or late male killing in *Drosophila*, depending on maternal host age.. Naturwissenschaften.

[36] Boyle L, O'Neill SL, Robertson HM, Karr TL (1993). Interspecific and intraspecific horizontal transfer of *Wolbachia* in *Drosophila*.. Science.

[37] Breeuwer JAJ, Werren JH (1993). Cytoplasmic incompatibility and bacterial density in *Nasonia vitripennis*.. Genetics.

[38] Bressac C, Rousset F (1993). The reproductive incompatibility system in *Drosophila simulans*: DAPI-staining analysis of the *Wolbachia* symbionts in sperm cysts.. J Invertebr Pathol.

[39] Clancy DJ, Hoffmann AA (1998). Environmental effects on cytoplasmic incompatibility and bacterial load in *Wolbachia* infected *Drosophila simulans*.. Entomol Exp Appl.

[40] Sinkins SP, Braig HR, O'Neill SL (1995). *Wolbachia pipientis*: bacterial density and unidirectional cytoplasmic incompatibility between infected populations of *Aedes albopictus*.. Exp Parasitol.

[41] Tortosa P, Charlat S, Labbé P, Dehecq JS, Barré H (2010). *Wolbachia* age-sex-specific density in *Aedes albopictus*: a host evolutionary response to cytoplasmic incompatibility?. PLoS One.

[42] Sakaguchi B, Poulson DF (1963). Interspecific transfer of the “sex-ratio”condition from *Drosophila willistoni* to *D. melanogaster*.. Genetics.

[43] Tsuchiyama S, Sakaguchi B, Oishi K (1978). Analysis of gynandromorph survivals in *Drosophila melanogaster* infected with the male-killing SR organisms.. Genetics.

[44] Sugimoto TN, Fujii T, Kayukawa T, Sakamoto H, Ishikawa Y (2010). Expression of a doublesex homologue is altered in sexual mosaics of *Ostrinia scapulalis* moths infected with *Wolbachia*.. Insect Biochem Mol Biol.

[45] Charlat S, Davies N, Roderick GK, Hurst GGD (2007). Disrupting the timing of *Wolbachia*-induced male-killing.. Biol Lett.

[46] Bopp D, Bell LR, Cline TW, Schedl P (1991). Developmental distribution of female specific *Sex-lethal* proteins in *Drosophila melanogaster*.. Genes Dev.

[47] Belmont AS (2006). Mitotic chromosome structure and condensation.. Curr Opin Cell Biol.

[48] Ivanovska I, Orr-Weaver TL (2006). Histone modifications and the chromatin scaffold for meiotic chromosome architecture.. Cell Cycle.

[49] Ferrée PM, Barbash DA (2009). Species-specific heterochromatin prevents mitotic chromosome segregation to cause hybrid lethality in *Drosophila*.. PLoS Biol.

[50] Lassy CW, Karr TL (1996). Cytological analysis of fertilization and early embryonic development in incompatible crosses of *Drosophila simulans*.. Mech Dev.

[51] Callaini G, Dallai R, Riparbelli MG (1997). *Wolbachia*-induced delay of paternal chromatin condensation does not prevent maternal chromosomes from entering anaphase in incompatible crosses of *Drosophila simulans*.. J Cell Sci.

[52] Harris HL, Braig HR (2003). Sperm chromatin remodeling and *Wolbachia*-induced cytoplasmic incompatibility in *Drosophila*.. Biochem Cell Biol.

[53] Tram U, Ferrée PM, Sullivan W (2003). Identification of *Wolbachia*-host interacting factors through cytological analysis.. Microbes Infect.

[54] Jaenike J (2007). Spontaneous emergence of a new *Wolbachia* phenotype.. Evolution.

[55] Tremethick DJ (2007). Higher-order structures of chromatin: the elusive 30 nm fiber.. Cell.

[56] Luger K, Hansen JC (2005). Nucleosome and chromatin fiber dynamics.. Curr Opin Struct Biol.

[57] Woodcock CL, Ghosh RP (2010). Chromatin higher-order structure and dynamics.. Cold Spring Harb Perspect Biol.

[58] Landmann F, Orsi GA, Loppin B, Sullivan W (2009). *Wolbachia* mediated cytoplasmic incompatibility is associated with impaired histone deposition in the male pronucleus.. PLoS Pathog.

[59] Raff JW, Glover DM (1988). Nuclear and cytoplasmic mitotic cycles continue in *Drosophila* embryos in which DNA synthesis is inhibited with aphidicolin.. J Cell Biol.

[60] Gonzalez C, Saunders RD, Casal J, Molina I, Carmena M (1990). Mutations at the asp locus of *Drosophila* lead to multiple free centrosomes in syncytial embryos, but restrict centrosome duplication in larval neuroblasts.. J Cell Sci.

[61] Yasuda GK, Baker J, Schubiger G (1991). Independent roles of centrosomes and DNA in organizing the *Drosophila* cytoskeleton.. Development.

[62] Debec A, Kalpin RF, Daily DR, McCallum PD, Rothwell WF (1996). Live analysis of free centrosomes in normal and aphidicolin-treated *Drosophila* embryos.. J Cell Biol.

[63] Zhang G, Breuer M, Förster A, Egger-Adam D, Wodarz A (2009). Mars, a *Drosophila* protein related to vertebrate HURP, is required for the attachment of centrosomes to the mitotic spindle during syncytial nuclear divisions.. J Cell Sci.

[64] Wilson PG, Borisy GG (1998). Maternally expressed gamma Tub37CD in *Drosophila* is differentially required for female meiosis and embryonic mitosis.. Dev Biol.

[65] Callaini G, Riparbelli MG, Dallai R (1994). The distribution of cytoplasmic bacteria in the early *Drosophila* embryo is mediated by astral microtubules.. J Cell Sci.

[66] Kose H, Karr TL (1995). Organization of *Wolbachia pipientis* in the *Drosophila* fertilized egg and embryo revealed by an anti-*Wolbachia* monoclonal antibody.. Mech Dev.

[67] Vaizel-Ohayon D, Schejter ED (1999). Mutations in *centrosomin* reveal requirements for centrosomal function during early *Drosophila* embryogenesis.. Curr Biol.

