# Supplementary material for: Wolbachia-Mediated Male Killing Is Associated with Defective Chromatin Remodeling
Source: PLoS One. 2012 Jan 23;7(1):e30045. doi: 10.1371/journal.pone.0030045 (PMC3264553; doi:10.1371/journal.pone.0030045)
Supplement: Table S1 — Developmental defects observed in early embryos obtained by Drosophila bifasciata KOS10 females. (DOC) [file pone.0030045.s001.doc]

**Table S1.** Developmental defects observed in early embryos obtained by *Drosophila bifasciata* KOS10 females.

| **Class^(1)^** | **n^(2)^** | **Meiosis (%)** | **Pronuclear apposition (%)** | **First mitosis** | | **Second mitosis** | | **Intravitelline mitoses** | | **Early arrested embryos^(4)^ (%)** | **Unfertilized eggs^(5)^ (%)** |
| --- | --- | --- | --- | --- | --- | --- | --- | --- | --- | --- | --- |
|  |  |  |  | **N^(3)^ (%)** | **D^(3)^ (%)** | **N (%)** | **D (%)** | **N (%)** | **D (%)** |  |  |
| 30-45 | 221 | 35 (15.8) | 25 (11.3) | 100 (45.4) | 24 (10.8) |  |  |  |  |  | 37 (16.7) |
| 60-90 | 270 |  |  |  |  | 39 (14.4) | 7 (2.6) | 94 (34.8) | 55 (20.4) | 30 (11.1) | 45 (16.7) |

^(1)^ Time of development after egg deposition (minutes).

^(2)^ Total number of eggsand/or embryos scored.

^(3)^ N, normal; D, defective.

^(4)^ Embryos with a variable number of abnormal spindles (monoastral or biastral). Sperm tail is present.

^(5)^ Eggs with a few barrel shaped anastral spindles. Sperm tail is absent.
