# Supplementary material for: Wolbachia-Mediated Male Killing Is Associated with Defective Chromatin Remodeling
Source: PLoS One. 2012 Jan 23;7(1):e30045. doi: 10.1371/journal.pone.0030045 (PMC3264553; doi:10.1371/journal.pone.0030045)
Supplement: Table S2 — Developmental defects observed in late embryos obtained by Drosophila bifasciata KOS10 females. (DOC) [file pone.0030045.s002.doc]

**Table S2.** Developmental defects observed in late embryos obtained by *Drosophila bifasciata* KOS10 females.

| **Class^(1)^** | **n^(2)^** | **Syncytial blastoderm** | | **Cellularization** | | **Gastrulation** | | **Germ band** | | **Early arrested embryos^(4)^ (%)** | **Unfertilized eggs^(5)^ (%)** |
| --- | --- | --- | --- | --- | --- | --- | --- | --- | --- | --- | --- |
|  |  | **N ^(3)^ (%)** | **D ^(3)^ (%)** | **N (%)** | **D (%)** | **N (%)** | **D (%)** | **N (%)** | **D (%)** |  |  |
| 3 | 336 | 142 (42.3%) | 91 (27.1%) |  |  |  |  |  |  | 40 (11.9%) | 63 (18.7%) |
| 4-5 | 282 | 13 (4.6%) | 8 (2.8%) | 85 (30.1%) | 54 (19.1%) | 24 (8.5%) | 17 (6.0%) |  |  | 28 (9.9%) | 53 (18.8%) |
| 10-20 | 292 |  |  | 6 (2.1%) | 3 (1.0%) | 10 (3.4%) | 7 (2.4%) | 112 (38.4%) | 72 (24.7%) | 28 (9.5%) | 54 (18.5%) |

^(1)^ Time of development after egg deposition (hours).

^(2)^ Total number of eggs and/or embryos scored.

^(3)^ N, normal; D, defective.

^(4)^ Embryos with a variable number of scattered abnormal spindles (monoastral or biastral). Sperm tail is present.

^(5)^ Eggs with a few barrel shaped anastral spindles. Sperm tail is absent.
