# Supplementary material for: Wolbachia-Mediated Male Killing Is Associated with Defective Chromatin Remodeling
Source: PLoS One. 2012 Jan 23;7(1):e30045. doi: 10.1371/journal.pone.0030045 (PMC3264553; doi:10.1371/journal.pone.0030045)
Supplement: Table S3 — Anti-Sxl staining of embryos collected 4–20 hours AED by Drosophila bifasciata KOS10 females. (DOC) [file pone.0030045.s003.doc]

**Table S3.** Anti-Sxl staining of embryos collected 4-20 hours AED by *Drosophila bifasciata* KOS10 females.

|  | **Sxl+** | **Sxl-** | | |
| --- | --- | --- | --- | --- |
| **n^(1)^** | **Post-blastoderm (%)** | **Post-blastoderm (%)** | **Early arrested embryos^(2 )^ (%)** | **Unfertilized eggs^(3)^ (%)** |
| 197 | 81 (41%) | 63 (32%) | 17 (8.6%) | 36 (18.6%) |

^(1)^ Total number of eggs and/or embryos scored.

^(2)^ Embryos with a variable number of abnormal spindles (monoastral or biastral). Sperm tail is present.

^(3)^ Eggs with a few barrel shaped anastral spindles. Sperm tail is absent.
